# Supplementary material for: Spatial patterns of tuberculosis and HIV co-infection in Ethiopia
Source: PLoS One. 2019 Dec 5;14(12):e0226127. doi: 10.1371/journal.pone.0226127 (PMC6894814; doi:10.1371/journal.pone.0226127)
Supplement: S1 Table — (DOCX) [file pone.0226127.s001.docx]

**Table S1:** Summary of independent variables, sources of data and definition of variables

| **Independent variables** | **Data sources** | **Definition** |
| --- | --- | --- |
| Socio-economic factors | | |
| Low wealth index | EDHS 2016 | Total number of people with low wealth index (poorer and poorest) divided by the total number of people participated in the survey. |
| Average number of persons per room | Ethiopia Atlas of Population Density | Average number of people living in a room |
| Unemployed population | Ethiopia Atlas of Population Density | Percentage of people in the labour force who were unemployed |
| Adult literacy rate | Ethiopia Atlas of Population Density | Percentage of population aged 15 years and above who had attended higher than secondary school or who can read and write a short simple statement |
| Behavioural factors | | |
| Chat chewing | EDHS 2016 | Total number of people chewing chat in the last one month prior to the survey divided by the total number of people participating in the survey |
| Alcohol drinking | EDHS 2016 | Total number of people drinking alcohol in the month prior to the survey divided by the total number of people participating in the survey |
| Health care access and knowledge and attitude regarding TB | | |
| Health care access problem | EDHS 2016 | Difficulty of getting advice or treatment due to lack of money, or distance to a health facility |
| Poor knowledge toward TB | EDHS 2011 | Number of people with poor knowledge towards TB divided by the total number of people participating in the survey. |
| Poor attitude towards TB | EDHS 2011 | Number of people with poor attitude towards TB divided by the total number of people participating in the survey |
| Proximity to international boarder | EDHS Spatial Analysis data | Straight-line distance to the nearest international border measured by meters |
| Climatic and environmental factors | | |
| Enhanced vegetation index | EDHS Spatial Analysis data | The average enhanced vegetation index which is calculated by measuring the density of green leaves in the near-infrared and visible bands. |
| Rainfall | EDHS Spatial Analysis data | Annual mean rainfall (mm) |
| Aridity | EDHS Spatial Analysis data | The average aridity index calculated by dividing the actual evapotranspiration by the potential evapotranspiration. |
| Mean temperature | EDHS Spatial Analysis data | Annual mean environmental air temperature (°C). |
