# Supplementary material for: Spatial patterns of tuberculosis and HIV co-infection in Ethiopia
Source: PLoS One. 2019 Dec 5;14(12):e0226127. doi: 10.1371/journal.pone.0226127 (PMC6894814; doi:10.1371/journal.pone.0226127)
Supplement: S2 Table — (DOCX) [file pone.0226127.s002.docx]

**Table S2:** The prevalence of TB, HIV and TB/HIV co-infection in Ethiopia at country, region, and zone levels, between June 2016 and June 2017.

|  |  | **Population prevalence of TB notification** | **Population prevalence of HIV infection** | **TB prevalence among HIV patients** | **HIV prevalence among TB patients** |  |
| --- | --- | --- | --- | --- | --- | --- |
|  | **Ethiopia** | 0.2 | 1.7 | 0.8 | 7.4 |  |
|  | **Addis Ababa** | 0.4 | 10.2 | 1.0 | 22.5 |  |
|  | **Afar** | 0.1 | 0.1 | 8.5 | 8.1 |  |
|  | Zone 01 | 0.1 | 0.0 | 6.6 | 3.5 |  |
|  | Zone 02 | 0.1 | 0.0 | 14.3 | 23.5 |  |
|  | Zone 03 | 0.0 | 0.0 | 11.4 | 5.2 |  |
|  | Zone 04 | 0.2 | 0.4 | 6.7 | 10.6 |  |
|  | Zone 05 | 0.2 | 0.1 | 7.5 | 8.2 |  |
|  | **Amhara** | 0.2 | 3.4 | 0.6 | 8.2 |  |
|  | South Gondar | 0.2 | 2.3 | 0.5 | 4.6 |  |
|  | North Wello | 0.2 | 4.4 | 0.4 | 11.9 |  |
|  | Oromiya Special zone | 0.3 | 3.9 | 0.6 | 8.4 |  |
|  | East Gojjam | 0.2 | 2.8 | 0.7 | 9.4 |  |
|  | North Gondar | 0.2 | 2.7 | 1.3 | 8.8 |  |
|  | South Wello | 0.2 | 5.6 | 0.4 | 9.7 |  |
|  | North Shewa | 0.2 | 2.0 | 0.7 | 8.4 |  |
|  | West Gojjam | 0.2 | 1.9 | 0.6 | 5.1 |  |
|  | Waghemira | 0.2 | 1.2 | 0.7 | 4.1 |  |
|  | Awi | 0.2 | 2.8 | 1.0 | 5.4 |  |
|  | Bahir Dar Special | 0.4 | 20.4 | 0.2 | 17.0 |  |
|  | Argoba Special | 0.1 | 0.2 | 33.3 | 14.7 |  |
|  | **Beneshangul Gumu** | 0.1 | 0.2 | 7.7 | 6.3 |  |
|  | Metekel | 0.2 | 0.5 | 10.0 | 5.6 |  |
|  | Kamashi | 0.0 | 0.0 | 8.2 | 5.3 |  |
|  | Assosa | 0.0 | 0.2 | 1.4 | 4.9 |  |
|  | Pawe Special | 0.0 | 0.3 | 1.8 | 4.2 |  |
|  | Maokomo Special | 0.1 | 0.0 |  | 23.3 |  |
|  | **Dire Dawa** | 0.7 | 8.7 | 1.0 | 12.6 |  |
|  | **Gambela** | 0.2 | 0.3 | 9.6 | 20.8 |  |
|  | Agnewak | 0.2 | 0.7 | 4.2 | 17.2 |  |
|  | Mejenger | 0.2 | 0.2 | 26.7 | 36.4 |  |
|  | Etang Special | 0.1 | 0.1 | 35.9 | 17.4 |  |
|  | Nuwer | 0.1 | 0.1 | 15.3 | 8.2 |  |
|  | **Harer** | 0.4 | 6.0 | 0.9 | 8.9 |  |
|  | **Oromiya** | 0.2 | 1.1 | 0.7 | 4.8 |  |
|  | Jimma | 0.2 | 0.3 | 2.0 | 1.9 |  |
|  | Arsi | 0.2 | 1.2 | 0.7 | 4.1 |  |
|  | East Wellega | 0.2 | 1.9 | 0.4 | 3.6 |  |
|  | Illu Aba Bora | 0.1 | 0.4 | 1.4 | 4.0 |  |
|  | West Wellega | 0.2 | 0.3 | 0.7 | 1.4 |  |
|  | West Shewa | 0.2 | 0.9 | 0.7 | 4.9 |  |
|  | East Shewa | 0.3 | 3.8 | 0.7 | 10.3 |  |
|  | North Shewa | 0.2 | 0.6 | 1.4 | 5.7 |  |
|  | West Hararge | 0.2 | 0.2 | 1.9 | 2.5 |  |
|  | East Hararge | 0.2 | 0.1 | 2.8 | 0.8 |  |
|  | Guji | 0.2 | 0.5 | 1.5 | 8.1 |  |
|  | Adama Special | 0.6 | 27.4 | 0.4 | 22.8 |  |
|  | Bale | 0.2 | 0.2 | 1.1 | 1.9 |  |
|  | Jimma Spe Town | 0.6 | 10.2 | 0.3 | 15.0 |  |
|  | West Arsi | 0.2 | 0.8 | 1.0 | 3.5 |  |
|  | Kelem Wellega | 0.2 | 0.4 | 0.9 | 1.8 |  |
|  | Horo Gudru Wellega | 0.2 | 1.5 | 0.7 | 4.7 |  |
|  | Borena | 0.2 | 0.2 | 3.1 | 5.0 |  |
|  | South West Shewa | 0.2 | 1.0 | 0.9 | 6.0 |  |
|  | Burayu Special | 0.4 | 15.0 | 0.4 | 13.9 |  |
|  | **SNNP** | 0.1 | 0.2 | 1.6 | 3.8 |  |
|  | Gurage | 0.2 | 0.6 | 0.8 | 3.2 |  |
|  | Hadiya | 0.2 | 0.1 | 2.6 | 2.9 |  |
|  | Sidama | 0.1 | 0.1 | 1.7 | 2.7 |  |
|  | Gedeo | 0.2 | 0.2 | 2.6 | 6.1 |  |
|  | Gamo Gofa | 0.1 | 0.3 | 1.3 | 5.1 |  |
|  | Kembata Tembaro | 0.1 | 0.2 | 1.1 | 4.8 |  |
|  | Wolayita | 0.1 | 0.2 | 0.9 | 3.6 |  |
|  | South Omo | 0.1 | 0.2 | 1.4 | 6.4 |  |
|  | Keffa | 0.1 | 0.2 | 1.6 | 4.3 |  |
|  | Sheka | 0.2 | 2.4 | 0.7 | 8.7 |  |
|  | Bench Maji | 0.1 | 0.4 | 2.3 | 5.7 |  |
|  | Yem Special | 0.5 | 0.8 | 0.1 | 3.1 |  |
|  | Amaro Special | 0.1 | 0.0 | 5.6 | 4.3 |  |
|  | Burji Special | 0.1 | 0.0 | 0.0 | 1.5 |  |
|  | Konso Special | 0.0 | 0.0 | 16.7 | 4.4 |  |
|  | Derashe Lyiu | 0.2 | 0.3 | 1.7 | 3.6 |  |
|  | Dawro | 0.0 | 0.0 | 2.1 | 2.4 |  |
|  | Basketo | 0.0 | 0.0 | 20.0 | 17.4 |  |
|  | Konta | 0.1 | 0.2 | 3.5 | 8.2 |  |
|  | Siliti | 0.1 | 0.3 | 0.7 | 2.0 |  |
|  | Alaba | 0.2 | 0.3 | 1.4 | 2.2 |  |
|  | Awassa Town | 0.1 | 0.3 | 2.0 | 4.4 |  |
|  | **Somali** | NA | NA | 14.5 | 5.5 |  |
|  | Afder | 0.0 | 0.0 |  | 15.0 |  |
|  | Korahe | 0.0 | 0.0 |  | 14.9 |  |
|  | Gode | 0.0 | 0.0 |  | 2.6 |  |
|  | Warder | 0.0 | 0.0 |  |  |  |
|  | Degehabur | 0.0 | 0.0 | 19.4 | 12.6 |  |
|  | Jijiga | 0.1 | 0.0 | 7.0 | 4.8 |  |
|  | Fik | 0.2 | 0.1 | 1.2 | 0.9 |  |
|  | Shinile | 0.0 | 0.0 |  | 5.2 |  |
|  | Liben | 0.0 | 0.0 |  | 57.1 |  |
|  | Shinile | 0.0 | 0.0 |  | 5.2 |  |
|  | **Tigray** | 0.2 | 1.9 | 0.9 | 8.4 |  |
|  | North Western Tigray | 0.1 | 1.5 | 0.7 | 5.3 |  |
|  | Eastern Tigray | 0.2 | 2.1 | 0.7 | 9.3 |  |
|  | Central Tigray | 0.1 | 0.7 | 0.8 | 4.2 |  |
|  | South East | 0.1 | 1.6 | 0.5 | 6.0 |  |
|  | South Tigray | 0.2 | 2.9 | 0.8 | 12.3 |  |
|  | Western Tigray | 0.3 | 1.8 | 1.8 | 6.6 |  |
|  | Mekele Especial Zone | 0.3 | 4.0 | 1.3 | 16.4 |  |
|  |  | | | | | |
