# Supplementary figures and images for: Spatial patterns of tuberculosis and HIV co-infection in Ethiopia
Source: PLoS One. 2019 Dec 5;14(12):e0226127. doi: 10.1371/journal.pone.0226127 (PMC6894814; doi:10.1371/journal.pone.0226127)

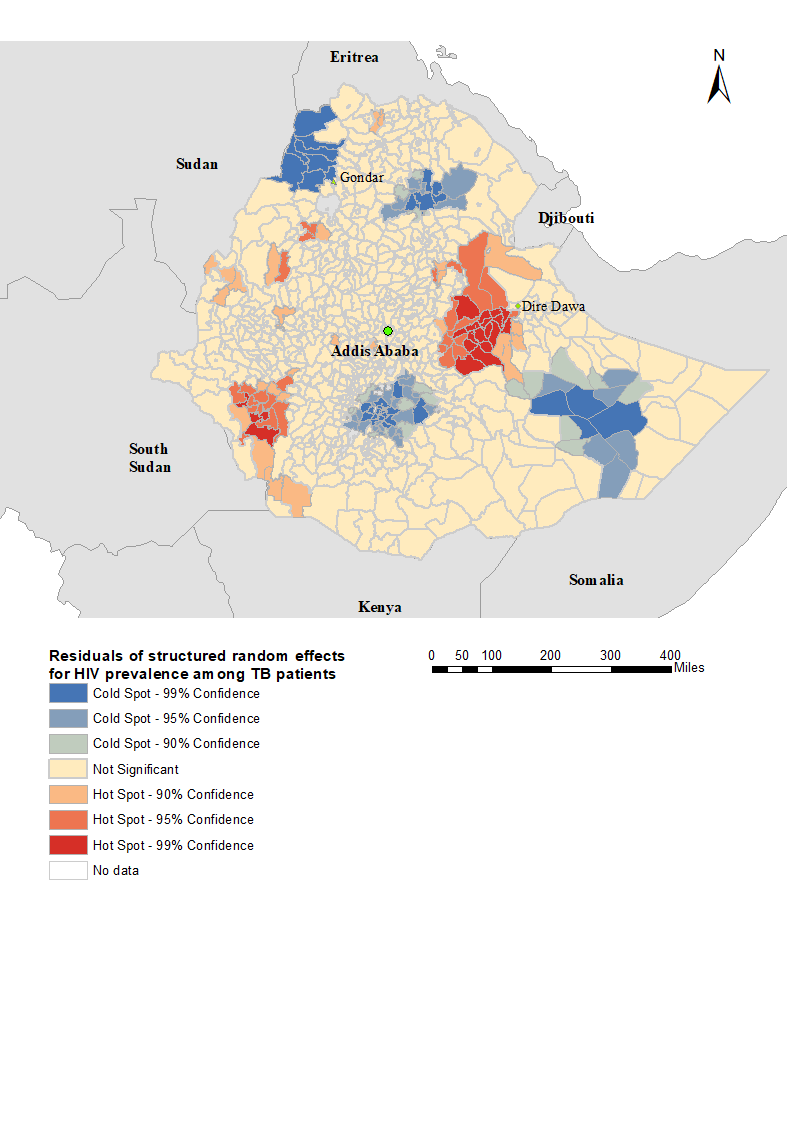

Supplement: S1 Fig — (TIF) [file pone.0226127.s003.tif]

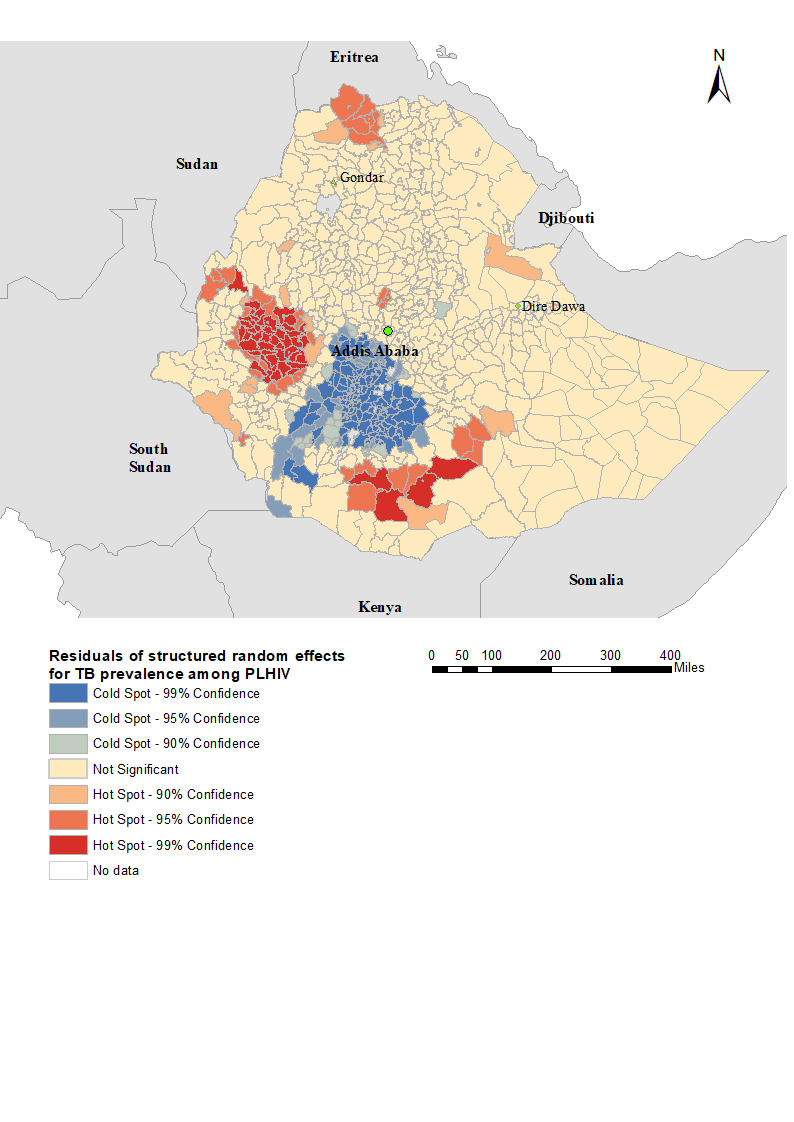

Supplement: S2 Fig — (TIF) [file pone.0226127.s004.tif]
