# Supplementary material for: Spatial patterns of tuberculosis and HIV co-infection in Ethiopia
Source: PLoS One. 2019 Dec 5;14(12):e0226127. doi: 10.1371/journal.pone.0226127 (PMC6894814; doi:10.1371/journal.pone.0226127)
Supplement: Attachment — Submitted filename: PONE-D-19-28080_reviewer.docx [file pone.0226127.s005.docx]

**Comments to the authors:**

Tuberculosis (TB) is the leading cause of death for individuals infected with Human immunodeficiency virus (HIV). Conversely, HIV is the most important risk factor in the progression of TB from the latent to the active status. In order to manage this double epidemic situation, an integrated approach that includes HIV management in TB patients was proposed by the World Health Organization and was implemented in many countries world-wide. To enable targeted intervention using the integrated approach, areas with high disease prevalence rates for TB and HIV need to be identified. The authors of the manuscript identified the hot and cold spot regions of HIV/TB co-infection in the districts of Ethiopia. They used a large data set to get inference on HIV/TB co-infection. In this regard, particularly in Ethiopia, the data regarding the HIV/TB co-infection are scare. This study successfully identified co-infection hot spots and associated ecological variables. The study could be used in the future to effectively manage HIV/TB co-infection in the Ethiopia. The present study is well designed, executed and the inferences drawn are well supported by the data. The manuscript is well written and understandable. Although, the concept of the study is not new, but could provide a reference for the management of TB/HIV co-infection in Ethiopia. The manuscript could be accepted for publication after incorporating the given suggestions.

**Abstract:**

**1.** The authors only mentioned hot spots for TB/HIV or HIV/TB con-infection in the districts of Ethiopia. They are advised to include cold spot regions in the abstract to distinguish between highly prevalent regions of co-infection to the least one.

**Introduction:**

**2.** The authors should also include in the introduction section, the earlier studies reporting the spatial distribution of co-infection of HIV/TB in other countries/regions. For eg. Aturinde A et al. 2019, Peres DA et al. 2019, Wei W et al. 2016.

**Results:**

**3.** The authors should include Figure S1 and Figure S2 in the main manuscript.

**4.** Supplementary Table S2 was incorrectly labeled as supplementary table S1. The authors should correct the labeling mistake.
